# Regulated Control of the Assembly and Diversity of LPS by Noncoding sRNAs

**DOI:** 10.1155/2015/153561

**Published:** 2015-11-05

**Authors:** Gracjana Klein, Satish Raina

**Affiliations:** Unit of Bacterial Genetics, Gdansk University of Technology, Narutowicza 11/12, 80-233 Gdansk, Poland

## Abstract

The outer membrane (OM) of Gram-negative bacteria is asymmetric due to the presence of lipopolysaccharide (LPS) facing the outer leaflet of the OM and phospholipids facing the periplasmic side. LPS is essential for bacterial viability, since it provides a permeability barrier and is a major virulence determinant in pathogenic bacteria. In *Escherichia coli*, several steps of LPS biosynthesis and assembly are regulated by the RpoE sigma factor and stress responsive two-component systems as well as dedicated small RNAs. LPS composition is highly heterogeneous and dynamically altered upon stress and other challenges in the environment because of the transcriptional activation of RpoE regulon members and posttranslational control by RpoE-regulated Hfq-dependent RybB and MicA sRNAs. The PhoP/Q two-component system further regulates Kdo_2_-lipid A modification via MgrR sRNA. Some of these structural alterations are critical for antibiotic resistance, OM integrity, virulence, survival in host, and adaptation to specific environmental niches. The heterogeneity arises following the incorporation of nonstoichiometric modifications in the lipid A part and alterations in the composition of inner and outer core of LPS. The biosynthesis of LPS and phospholipids is tightly coupled. This requires the availability of metabolic precursors, whose accumulation is controlled by sRNAs like SlrA, GlmZ, and GlmY.

## 1. Introduction

Extensive studies in the last decade have established beyond doubt that, in addition to known roles for DNA-binding transcriptional regulators, noncoding regulatory RNAs play a pivotal role in several key aspects of bacterial physiology. As most of such RNAs are small in size and majority of them are noncoding RNAs, they are commonly referred as small noncoding RNAs (sRNAs). They contribute in the regulation of important processes including nutrient uptake, transport of specific substrates, maintenance of metabolic fluxes, iron homeostasis, carbon metabolism, stress responses, biofilm formation, host-cell contact, and homeostatic control of key components of the cell envelope (reviewed in [1–3]). Regulatory RNAs can act by different mechanisms, modulating gene expression either positively or negatively. One class of regulatory RNAs comprises riboswitches or RNA thermometers, which are part of the same mRNA that they regulate [4–6]. Their leader sequence in the 5′ UTR of mRNA can adopt different conformations in response to either ligand binding or changes in nutritional status, or specific stresses such as temperature. Interaction with ligand or structural changes upon stress allow for a direct and immediate response. Another very well characterized group of regulatory RNAs acts by base-pairing with target mRNA(s) and modulates either their stability or their translation. Depending on their location with respect to their target, these molecules are designated to act as* cis-* or* trans*-acting RNAs [1–3]. Majority of them were initially identified to be located in the intergenic regions [7]. However, recently regulatory RNAs have also been identified in the 3′ UTR of mRNAs [8, 9]. Many of these sRNAs require the RNA chaperone Hfq for function. Hfq acts by facilitating base-pairing between sRNAs and target mRNAs [1–3]. Hfq is also required for stabilization of sRNAs by protecting them from RNA degradation machine [10]. Some regulatory RNAs can also bind specific protein as their target and modify protein activity or act by sequestration of substrate. Classical examples of such RNAs include* E*.* coli* CsrA binding CsrB-CsrC sRNAs and 6S RNA, which binds to housekeeping form of RNA polymerase E*σ* ^70^ [11, 12]. Tight regulation mediated by sRNAs is often the result of interconnected circuits acting in feedforward or feedback mechanisms that can regulate transcriptional factors and two-component systems constituting nodal controls of complex biological networks. Hence, Gram-negative bacteria, like* Escherichia coli*, encode over 100 sRNAs and several of them have been well characterized, regulating diverse cellular pathways [1–3].

In order to achieve high sensitivity, many sRNAs are transcribed in response to specific signals. The transcription of such sRNAs is often regulated by specialized sigma factors or by two-component systems. In turn, sRNAs may provide the feed-back mechanism for the downregulation of sigma factors and two-component systems once the signal is dealt with. Often downregulation by sRNAs is achieved by reducing the synthesis or translation of these regulators. This allows for the dampening of elevated stress responses or altered metabolic pathways.

**Figure 1:**
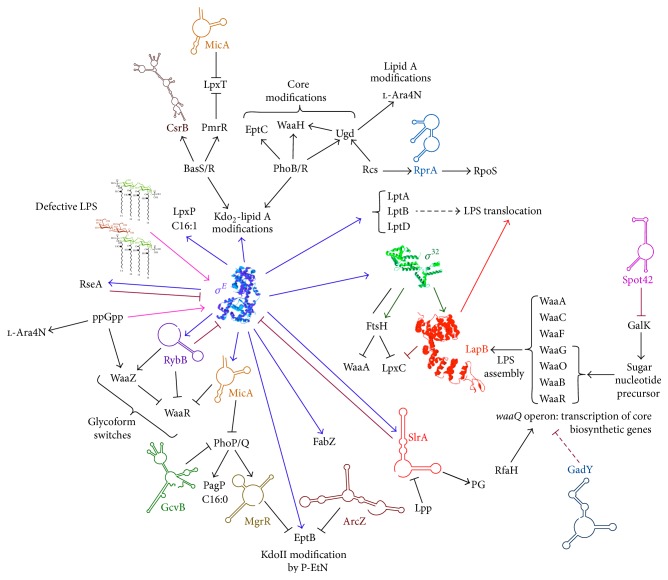
Regulatory networks controlling the LPS biosynthesis and nonstoichiometric modifications of lipid A and the LPS core. The key role mediated by RpoE-regulated noncoding sRNAs MicA, RybB, and SlrA with their major targets and how RpoE responds to LPS defects is depicted. RNA polymerase in complex with RpoE also transcribes some of the* lpt* genes, whose products are involved in the LPS translocation. RybB plays the major role in controlling the LPS composition by the translational repression of WaaR in RpoE-inducing conditions. RpoE in turn is subjected to a negative feedback regulation by RybB and SlrA that downregulate the synthesis of major cell envelope components to achieve homeostasis. RpoE also transcribes the* eptB* gene leading to the modification of KdoII by P-EtN. The translation of* eptB* mRNA is repressed by PhoP/Q-regulated MgrR sRNA. MicA and GcvB by base-paring repress the* phoP* mRNA translation. MicA substrate also includes* lpxT*, whose product mediates phosphorylation of lipid A generating triphosphorylated lipid A. Central roles played by BasS/R-dependent lipid A modifications and PhoB/R-dependent GlcUA and P-EtN incorporation in regulating the core biosynthesis are indicated. On the right side, proteolytic regulation of the first committed step in the LPS biosynthesis mediated by the RpoH-dependent FtsH/LapB complex is presented. LapB also couples the LPS synthesis and assembly with translocation system. Translation repression of* lpp* by SlrA regulates the availability of fatty acid pools for the synthesis of phospholipids. Spot42 sRNA regulates the availability of sugar nucleotide precursors for glycosyltransferases in response to the presence of either galactose or glucose by inhibiting the translation of* galK* within the* gal* operon.

One of the most well characterized stress responsive sigma factor is RpoE, which controls the transcription of several genes with a dedicated function in the envelope biogenesis (Figure 1). Thus, RpoE regulon members in* E*.* coli* include genes encoding periplasmic folding factors (*surA*,* fkpA*,* skp*, and* dsbC*), proteases [*degP*,* ecfE* (*rseP*)], components of outer membrane proteins (OMPs) assembly (*bamA-E*), genes whose products are involved in lipopolysaccharide (LPS) translocation and assembly (*lptA*,* lptB*, and* lptD*), synthesis of phospholipids and lipid A (*lpxP*,* lpxD*,* fabZ*,* lpxA*, and* lpxB*), and the* rpoH* gene encoding heat shock sigma factor [13]. RpoE positively autoregulates its own transcription and regulates its own activity negatively by transcribing genes (*rseA* and* rseB*), whose products act as negative regulators [14–16]. RpoE regulon also includes three sRNAs (RybB, MicA, and SlrA) [9, 17–22].

Induction of RpoE upon envelope stress or due to deletion of antisigma factor RseA leads to pronounced reduction in the amounts of OMPs, Lpp lipoprotein, and alterations in LPS composition [9, 22–25]. The repression of OMPs synthesis occurs at the posttranscriptional level, wherein base-paring sRNAs like MicA inhibits translation and decay of the* ompA* mRNA and RybB inhibits translation of* ompC*,* ompW,* and* ompN* mRNAs [17–19]. This inhibition in the synthesis of major OMPs under stress conditions is to maintain an envelope homeostasis, as some of these OMPs constitute major abundant proteins under normal growth conditions. Thus, RpoE-regulated sRNAs, MicA, RybB, and SlrA provide one of the best examples of the positive regulation (feedforward mechanism) by RpoE in response to envelope stress and RpoE downregulation by these sRNAs in a feedback mechanism (Figure 1) [9, 24]. The orchestrated activities of RpoE, MicA, RybB, and SlrA insure homeostatic control of outer membrane components such as LPS, most abundant lipoprotein Lpp and OMPs. Such a regulation also allows integration of diverse signals from two-component systems with the RpoE sigma factor. There exists an in-built link between OMP content and LPS structure. Severe defects in LPS trigger massive induction of RpoE and this can simply explain reduction in the amounts of OMPs due to their downregulation of synthesis by MicA- and RybB-mediated translational repression.

**Figure 2:**
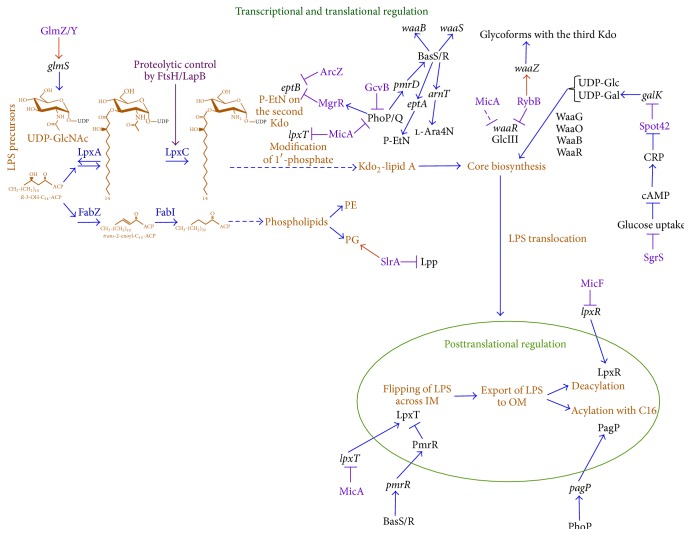
A flow chart depicting various steps regulated by noncoding sRNAs during the biosynthesis of LPS or its regulated nonstoichiometric modifications. The LPS biosynthesis begins with the GlmS-mediated synthesis of GlcN6P. GlcN6P serves as a precursor for UDP-GlcNAc, which is a metabolic intermediate for the LPS synthesis. UDP-GlcNAc is acylated by LpxA using* R*-3-hydroxymyristate, followed by deacylation by LpxC. The expression of the* glmS* mRNA is regulated by GlmZ/Y sRNAs at the posttranscriptional level, while the amount of LpxC is regulated by FtsH/LapB-mediated turnover. The balanced synthesis of LPS and phospholipids requires SlrA-dependent translational repression of Lpp to feed more fatty acids pools for the phospholipid synthesis. Kdo_2_-lipid A modifications are regulated by transcriptional induction of the BasS/R two-component system and RpoE-dependent induction of the* eptB* gene. PhoP/Q activation can amplify induction of the transcription of BasS/R-regulated* eptA*,* arnT*, and* pmrD* genes. The PhoP synthesis is negatively regulated by MicA and GcvB sRNAs at the translational level. The* eptB* subjected to translational repression by PhoP/Q-regulated MgrR. The LPS core biosynthesis could be further fine-tuned by regulating sugar uptake and the amount of UDP-Gal and UDP-Glc precursors, requiring SgrS and Spot42 sRNAs. The RpoE-dependent induction of RybB leads to the synthesis of LPS glycoforms with a third Kdo and truncation of the outer core due to translational repression of WaaR by RybB. After the completion of LPS synthesis and its flipping across the IM, lipid A can be modified by LpxT, whose activity is repressed at the posttranslational level by PmrR. After the incorporation of LPS in the outer membrane, lipid A may be further acylated by posttranslational activation of PagP or deacylated by LpxR. The LpxR synthesis is inhibited by MicF at its translational level by base-paring with the* lpxR* mRNA. Note that LpxR is absent in* E. coli* K-12 but is presented in several pathogenic* E. coli* strains and* Salmonella*. Other lipid A modifications not observed in* E. coli* K-12 are not shown.

The outer membrane of Gram-negative bacteria is composed of highly abundant OMPs and LPS. Approximately 2 × 10^6^ molecules of LPS cover nearly 75% of the cell surface [26]. Thus, cellular regulatory controls are in place to monitor the biogenesis of OMPs and LPS. In many Gram-negative bacteria, sRNAs have been shown to play an important role for the homeostasis of the envelope. In this review, specific sRNAs encoded in the* E. coli* genome that regulate the synthesis, composition, and structural modification of LPS, an essential component of the cell envelope, are highlighted. As will be evident, specific sRNAs control events in LPS biosynthesis from its early steps, regulating a balance between amounts of fatty acids (phospholipids) and LPS as well as incorporation of specific nonstoichiometric modifications in LPS. These structural alterations are critical for imparting resistance to antibiotics and survival under defined environmental niches (Table 1). Dedicated sRNAs most notably control the accumulation of specific LPS glycoforms, thereby controlling LPS composition and its heterogeneity (Figures 1, 2, and 5). The ability to generate glycoforms with truncations in the outer core of LPS is modulated by sRNAs and RpoE and is critical for the addition of O-antigen that confers serum resistance [25]. In pathogenic Gram-negative bacteria, LPS is known to be a major virulence factor. Consistent with the key role of LPS in bacterial virulence, the attenuated virulence phenotype of* hfq* mutants can be ascribed to LPS alterations that are regulated by Hfq-dependent sRNAs [1, 27].

## 2. LPS Biosynthesis and Its Heterogeneity

The cytoplasm of Gram-negative bacteria, such as* E. coli*, is surrounded by an inner membrane (IM) that separates an aqueous periplasmic compartment containing peptidoglycan from the outer membrane (OM). The OM is asymmetric in nature with phospholipids facing the inner leaflet and the LPS facing towards the outside. LPS is a complex glycolipid and constitutes the major amphiphilic component of the OM. Its chemical composition contributes to the permeability barrier function. The synthesis of LPS and phospholipids is tightly coregulated and held at a nearly constant ratio of 0.15 to 1.0 [26]. However, LPS is highly heterogeneous in composition and comprised of a mixture of different glycoforms [25, 28, 29]. The ratio of different glycoforms, which differ due to nonstoichiometric substitutions, is regulated by various stress-responsive two-component systems such as PhoB/R (sensing phosphate concentration), PhoP/Q (sensing divalent cations like Mg^2+^), and BasS/R (responsive to Fe^3+^, Zn^2+^, antimicrobial peptides) and above all by the sigma factor RpoE the master regulator of envelope biogenesis processes [25]. Embedded within these regulatory systems are several noncoding sRNAs (MicA, RybB, SlrA, MgrR, and ArcZ) that in turn regulate the incorporation of some of these nonstoichiometric modifications and hence control the LPS heterogeneity (Figures 1 and 2).

In spite of this heterogeneity, LPS in general shares a common architecture composed of a membrane-anchored phosphorylated and acylated *β*(1 → 6)-linked GlcN disaccharide, termed lipid A, to which a carbohydrate moiety of varying size is attached [30]. The latter may be divided into a proximal core oligosaccharide and, in smooth-type bacteria, a distal O-antigen.

The biosynthesis and translocation of LPS requires the function of more than 50 genes. Several of them are essential and unique to bacteria and hence they are excellent targets for identification of new inhibitors and the development of novel antibiotics. Biosynthesis of LPS is thought to occur on the* cis* side of the plasma membrane as some of the enzymes, like those involved in the early steps of lipid A biosynthesis are either integral IM proteins (LpxK, WaaA, LpxL, and LpxM) or peripheral IM-associated (LpxB and LpxH) or unstable cytoplasmic protein LpxC [30].

**Figure 3:**
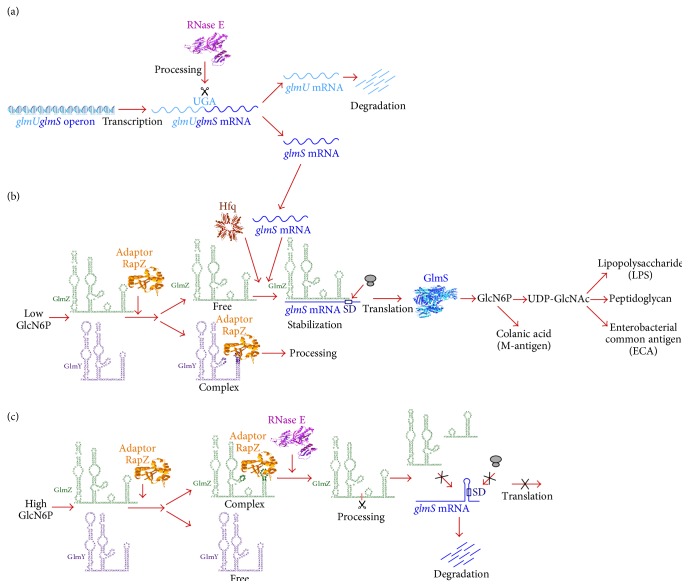
Hierarchical regulation of glucosoamine-6-phosphate synthase GlmS. (a) Genes* glmUS* constitute a bicistronic operon, which is cleaved by RNase E at the UGA stop codon of* glmU*, generating into monocistronic mRNAs that are rapidly degraded. (b) Under GlcN6P limiting conditions, the* glmS* mRNA base-pairs with intact GlmZ leading to stabilization of the* glmS* mRNA and also activation of its translation by disrupting inhibitory stem-loop structure that otherwise sequesters the Shine-Dalgarno (SD). This base pairing requires Hfq. Under GlcN6P limiting conditions, GlmZ processing is prevented by sequestration of the RapZ adaptor protein, preventing RapZ targeting to RNase E-mediated degradation of GlmZ. (c) When GlcN6P amounts are high, GlmY sRNA is present in low amounts. This allows the recruitment of RapZ to GlmZ leading to RNase E-mediated cleavage. This cleavage leads to the generation of a processed form of* glmZ* that lacks complementarity to* glmS* and hence inability to activate* glmS* translation due to inability to access SD. This also leads to a rapid degradation of the* glmS* mRNA.

The enzymatic activities of glucosamine-6-phosphate synthase GlmS and the deacylase LpxC control the biosynthetic branch points of major cell envelope components, using two key precursors molecules UDP-*N*-acetyl-d-glucosamine (UDP-GlcNAc) and* R*-3-hydroxymyristoyl acyl carrier protein (*R*-3-hydroxymyristoyl-ACP), respectively [30, 31] (Figure 2). Thus, the regulation of GlmS and LpxC is important and essential for bacterial cell envelope biogenesis. In these processes, specific sRNAs play key regulatory roles (Table 1). GlcN6P and its downstream product serve as the glucosamine source for the synthesis of peptidoglycan, lipid A, enterobacterial common antigen (ECA), and colanic acid (M-antigen) (Figures 2 and 3). ECA and M-antigen can be ligated to LPS by the WaaL ligase in the place of O-antigen [32, 33].

## 3. Homologous GlmY and GlmZ sRNAs Regulate the Synthesis of GlmS Required for the Biosynthesis of LPS Precursor UDP-GlcNAc

GlmS catalyzes the first step in hexosamine metabolism, converting fructose-6-phosphate (Fru6P) into glucosamine-6-phosphate (GlcN6P), which constitutes the first rate-limiting step in the synthesis of UDP-GlcNAc and hence in the LPS biosynthesis as well as in the other above mentioned constituents of the cell envelope (Figures 2 and 3). The conversion of GlcN6P into UDP-GlcNAc requires two additional essential enzymes: GlmM and GlmU [34]. GlmS synthesis is negatively regulated by its product GlcN6P (feedback inhibition) in a posttranscriptional manner [35].

In* E. coli*,* glmU* and* glmS* genes are transcribed as an operon and the corresponding primary transcript is cleaved by RNase E at the* glmU* stop codon UGA, rendering* glmU* transcripts unstable. The* glmS* mRNA is also unstable, unless its translation is activated by base-pairing with GlmZ sRNA [36]. Specifically, GlmZ activates translation of* glmS* through an anti-antisense mechanism (reviewed in [37, 38]). GlmZ acts on the* glmS* mRNA, whose ribosome-binding site is normally sequestered within a hairpin. In concert with the RNA chaperone Hfq, GlmZ base-pairs with* glmS* transcript and facilitates its translation by stabilizing* glmS* message and preventing the formation of inhibitory structure that occludes ribosome-binding site of* glmS* (Figure 3) [39]. The activity of GlmZ is controlled by its processing, due to which it can exist in two forms: the unprocessed form that activates* glmS* translation while the processed form is unable to bind the* glmS* mRNA (Figure 3). The processed shorter form of GlmZ lacks the* glmS* target site [36]. GlmZ and GlmY are structurally homologous sRNA. However, GlmY lacks the complementarity region to the* glmS* mRNA and stimulates GlmS synthesis by suppressing the GlmZ degradation by RNase E and RapZ (YhbJ). Thus, because of the structural similarity with GlmZ, GlmY functions by molecular mimicry and high levels of GlmY titrate RapZ from GlmZ and prevent its processing [38]. However, GlmZ processing is coupled with GlcN6P levels (see below).

In summary, it has been shown that the accumulation of GlmS is negatively regulated by RapZ and positively by two homologous sRNAs GlmY and GlmZ [35, 36, 38]. Mutations, causing defects in RapZ or increase in GlmY or GlmZ concentrations, lead to increased expression of* glmS*. Each of these sRNAs has a unique role and they work in a hierarchical feedback loop to activate* glmS* expression in response to intracellular levels of GlcN6P (Figure 3) [36, 38]. Under limiting GlcN6P conditions, homologous GlmY sRNA accumulates and sequesters RNase adaptor protein RapZ, preventing GlmZ processing [35, 40]. In contrast, at high concentrations of GlcN6P, GlmZ is preferably bound by RapZ and consequently degraded by RNase E (Figure 3). GlmY does not bind Hfq and hence GlmY functions as a molecular decoy acting as an antiadaptor titrating away GlmZ degradation system of the RNase E adaptor RapZ [36]. Hence, the synthesis of GlcN6P is tightly regulated in response to its synthesis demands and concentration sensing by GlmY/GlmZ sRNAs. Thus, it constitutes an early step in the synthesis of LPS precursor UDP-GlcNAc.

An interesting connection in the regulation of the transcription of* rapZ* and* glmY* with RpoE activation is likely and needs further studies. The* rapZ* gene is located within the* rpoN* operon. The transcription of the* rpoN* gene is positively regulated by RpoE and can also have an impact on its mRNA levels from RpoE-dependent transcription regulation of promoters located upstream of* lptA*/*B* genes [41]. Furthermore, RpoN and the two-component system QseF/E regulate the transcription of GlmY. Interestingly, QseF/E and its orthologs play critical roles in virulence of several enterobacterial pathogens like* Salmonella*,* Yersinia pseudotuberculosis*, and enterohemorrhagic* E. coli* (EHEC) [42]. Thus, these findings further underline the importance of sRNAs involvement like GlmY/S in other regulatory pathways that could impact not only LPS, but also other virulence factors.

## 4. Balanced Biosynthesis of LPS and Phospholipids and Feedback Control by sRNAs

### 4.1. Feedback Control by RpoE-Dependent SlrA sRNA

The biosynthesis of LPS begins with the acylation of UDP-GlcNAc with* R*-3-hydroxymyristate derived from* R*-3-hydroxymyristoyl-ACP [43].* R*-3-Hydroxymyristoyl-ACP also serves as a precursor for the synthesis of phospholipids [44]. The second reaction of the lipid A biosynthesis is catalyzed by LpxC [UDP-3-*O*-(*R*-3-hydroxymyristoyl)-*N*-acetylglucosamine deacetylase], constituting the first committed step in the LPS synthesis, as the equilibrium constant for the first reaction catalyzed by LpxA is unfavourable. Next, seven additional enzymes are required for the completion of synthesis of the minimal LPS structure Kdo_2_-lipid A, which acts as a substrate for further sequential incorporation of various sugars by specific glycosyltransferases (Figure 2).

The* fabZ* gene encodes the* R*-3-hydroxymyristoyl-ACP dehydratase, which catalyzes the first key step in the phospholipid biosynthesis. Thus, the fundamental common substrate of LpxC and FabZ,* R*-3-hydroxymyristoyl-ACP, comprises an essential branch point in the biosynthesis of phospholipids and the lipid A part of LPS (Figure 2). Hence, an* in vivo* competition of FabZ and LpxC for their common substrate sets a balance in the synthesis of phospholipids and lipid A [45]. High level of the LpxC accumulation is toxic to cells due to the excess of LPS over phospholipids and resulting depletion of the common substrate* R*-3-hydroxymyristoyl-ACP [9, 46]. Thus, regulation of LpxC amounts is crucial for this critical biosynthetic checkpoint. LpxC is an unstable protein and its turnover is mediated by the essential IM-located, Zn-dependent metalloprotease FtsH in conjunction with the recently identified heat shock protein LapB [9, 46, 47]. LapB is also an IM-anchored protein and contains TPR repeats in its N-terminal domain and a rubredoxin-like C-terminal domain, both of which were found to be essential for activity [9]. The absence of either LapB or FtsH is toxic as cells accumulate elevated levels of LPS with a phenomenon that can be compensated by suppressor mutations in the* fabZ* gene or by overexpression of* fabZ*, which restore a balance between phospholipids and LPS [9, 46, 47]. Alternatively, increasing the free fatty acid pools that serve as precursors for the phospholipid synthesis can also overcome the lethality in the absence of LapB (Figures 1 and 2) [9]. This finding was based on the isolation of multicopy suppressor identifying a new sRNA SlrA [9]. The SlrA-mediated multicopy suppression could be attributed to* lpp* (encoding Braun's lipoprotein) as a substrate of SlrA sRNA (Figures 1 and 2). Consistent with such a notion, transposon insertion in the* lpp* gene also restored* lapB* defects [9]. Thus, reduction in Lpp mediated by overexpression of SlrA sRNA or loss of* lpp* can bypass lethality, when the LPS synthesis is increased due to accumulation of LpxC. Lpp is the most abundant lipoprotein in* E*.* coli* (7 × 10^5^ molecules per cell) and contains three lipid chains. Hence, its depletion increases the pool of free fatty acids for the synthesis of phospholipids and helps to restore the balance with LPS. Indeed, the overexpression of SlrA sRNA, also called MicL, leads to repression of the Lpp synthesis and hence mimics a loss of function mutation in the* lpp* gene [9, 22]. SlrA/MicL was shown to inhibit* lpp* at its translational level by direct base-pairing in an Hfq-dependent manner.

*slrA* with its own promoter is encoded within the 3′ end of the coding region of the* cutC* gene [9, 22]. SlrA was found to be an 80 nt sRNA, which is synthesized as a 307 nt precursor mRNA that is processed. At present, the enzyme required for the processing of* slrA* mRNA is unknown. Interestingly, transcription of* slrA* is directed by E*σ* ^E^ polymerase and thus* slrA* is the third known sRNA of the RpoE regulon [9, 22]. SlrA expression also provides a feedback mechanism of control of RpoE activity, since overexpression of SlrA sRNA downregulates elevated envelope stress responses by monitoring Lpp amounts [9]. Indeed, overexpression of SlrA causes downregulation of DegP synthesis in (*lapA lapB*) mutants that is otherwise elevated in such backgrounds due to the RpoE induction [9]. Thus, repression of the* lpp* mRNA translation leading to restoration of phospholipid synthesis to counteract increase in LPS amount and the control of RpoE envelope stress response by a feedback mechanism is the main function of SlrA.

### 4.2. A Novel* cis*-Encoded sRNA May Control LpxC Amounts in* Pseudomonas aeruginosa*

Interestingly, SlrA sRNA and LapB are absent in* Pseudomonas aeruginosa* [9]. LpxC of* P. aeruginosa* is not a substrate of FtsH [48]. Examination of sRNAs in* P. aeruginosa* revealed the presence of an 84 nt sRNA PA4406, located in the intergenic region between* ftsZ* and* lpxC* genes. The existence of this sRNA has been verified experimentally and it is transcribed in the same sense as its potential target* lpxC* [49–51]. It is likely that this sRNA controls LpxC based on the repeated isolation of a mutation that confers resistance to novel LpxC inhibitor LpxC-4 [51, 52]. This mutation (C to A change) is located 11 nt upstream of the* lpxC* translational start site and maps to the 3′ end of the sRNA PA4406 [51]. Predicted structure suggests that this C to A mutation is located within the hairpin structure that pairs with G 18 nucleotide upstream of* lpxC* and could impact accessibility of ribosome-binding site. This mutation causes a 3-fold increase in* lpxC* expression, which could be as a result of increase in either the mRNA level or its differential turnover. The existence of such regulatory sRNA reflects an additional role for RNAs in this important pathogenic bacterium in regulating LpxC amounts and hence the LPS biosynthesis. The location of this* cis*-acting 84 nt sRNA in* P. aeruginosa* in the same sense as its putative target is rather rare. Most of the* cis*-acting sRNAs are located on the opposite strand of their target [1–3]. Further, most of the LPS regulating sRNAs, like MicA, RybB, SlrA, and MgrR, are* trans*-acting, thus providing another interesting dimension to this PA4406 sRNA in* P. aeruginosa*. However, it is not clear if PA4406 sRNA arises due to transcriptional processing or if it has its own promoter.

## 5. Biosynthesis of LPS and Its Regulated Modifications

Lipid A and Kdo are the most conserved elements of LPS between species. In* E. coli*, a bisphosphorylated lipid A precursor molecule, termed lipid IV_A_, is synthesized from UDP-GlcNAc, following six distinct enzymatic reactions. The lipid IV_A_ precursor serves as an acceptor for the addition of two Kdo residues mediated by the bifunctional enzyme Kdo transferase WaaA. This reaction results in the synthesis of the Kdo_2_-lipid IV_A_ intermediate. This intermediate serves as a substrate for late acylation reactions catalyzed by LpxL (lauroyl transferase) and LpxM (myristoyl transferase) to generate hexa-acylated lipid A (Kdo_2_-lipid A) and also for various glycosyltransferases for the completion of core biosynthesis. However, at low temperature (12°C) LpxP mediates the addition of palmitoleate chain (C16:1) in ~80% of LPS molecules in the same place where lauroyl residue is usually present [53]. The* lpxP* gene is RpoE-regulated at the transcriptional level (Figure 1). The incorporation of palmitoleate in place of laurate suggests the presence of such unsaturated fatty acids aids in homeoviscous adaptation in response to cold stress or when LPS is composed of glycosylation free lipid IV_A_ derivatives as observed in* waaA* mutants [54].

**Figure 4:**
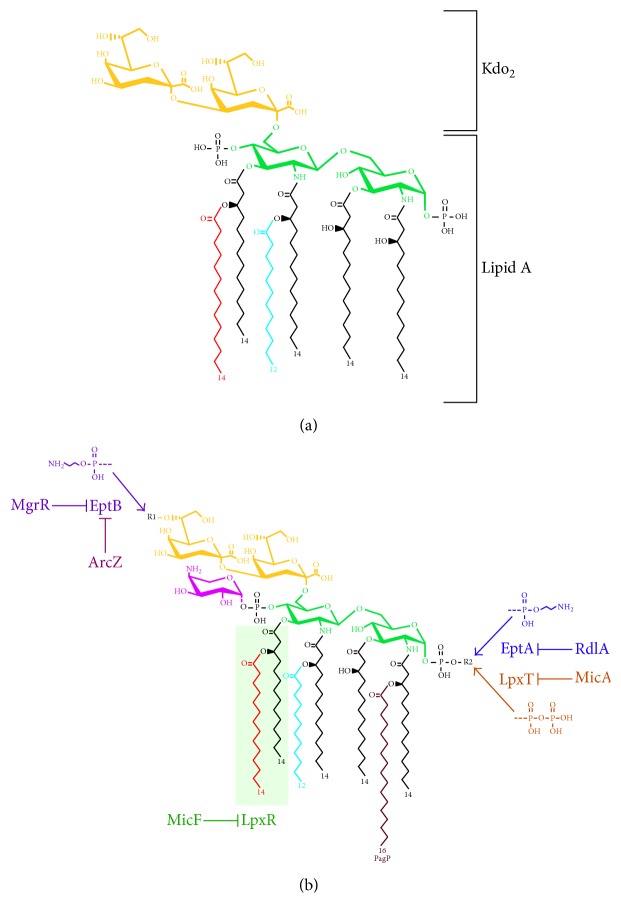
Chemical structures of unmodified Kdo_2_-lipid A (a) and modified Kdo_2_-lipid A derivatives (b). The synthesis of P-EtN transferase EptB is repressed by PhoP/Q-regulated MgrR sRNA. MicA and MicF repress LpxT and LpxR, respectively, by base-pairing of their mRNAs. RdlA sRNA is predicted to act on* eptA*; however impact on lipid A modification is not known.* R1* and* R2* indicate modification sites.

The Kdo_2_-lipid A constitutes the minimal LPS structure that is required for viability of bacteria like* E. coli* under standard laboratory growth conditions [54]. Interestingly, this structure is quite dynamic and is often subjected to nonstoichiometric modifications that involve activation of two-component systems and specific noncoding sRNAs (Figure 4). Some of these modifications are critical for resistance to cationic antimicrobial peptides and also for bacterial virulence in pathogenic bacteria. Most of the modifications involve either reducing the net negative charges of lipid A by modifying the 1 and 4′ ends of phosphate residues or the addition or removal of acyl chains (Figure 4). Similarly, modifications are also known to occur in the core region, like incorporation of phosphoethanolamine (P-EtN), uronic acids, or additional Kdo residues [25, 28].

Genes involved in the synthesis and modifications of lipid A and LPS are regulated by several transcriptional and posttranscriptional factors including RpoE, sRNAs that may be or not regulated by RpoE, and the two-component systems BasS/R (also known as PmrA/B), PhoP/Q, and PhoB/R in* E. coli* and* Salmonella*. (Figures 1 and 2). Additional two-component systems, like RcsB/C, EvgA/S, may further modulate one or more of these systems or induce a specific gene that is subjected to multiple transcriptional controls, for example, the* ugd* gene [28, 55]. It is known that RpoE regulon members include genes, whose products are involved in LPS biosynthesis, LPS transport, like* lpt* genes [13, 41], and LPS modification system controlled by the product of the* eptB* gene and MicA and RybB sRNAs [24, 25, 54, 56, 57] (Figures 1 and 2). MicA at the same time negatively regulates the two-component system PhoP/Q by the translational repression of PhoP [21]. To integrate signal transduction and cross talk between two-component systems and RpoE-dependent sRNAs, severe defects in LPS dramatically induce RpoE activity and this in turn is reflected in LPS composition and its modifications [25, 54].

In* E. coli* and* Salmonella enterica* serovar Typhimurium, BasS/R (PmrA/B) is induced upon exposure to low pH, excess of Fe^3+^, Zn^2+^, and Al^3+^, challenge by antimicrobial peptides, or treatment with the nonspecific phosphatase inhibitor ammonium metavanadate [28, 54, 58–60]. The most noticeable changes in lipid A involve the nonstoichiometric incorporation of P-EtN and 4-amino-4-deoxy-l-arabinose (l-Ara4N) residues by EptA and ArnT, respectively (Figure 4). EptA and ArnT transferases mediate the modification of 1-phosphate and 4′-phosphate by P-EtN and l-Ara4N, respectively. Covalent modifications by l-Ara4N and P-EtN cause decrease in the overall negative charge, which is essential for resistance to cationic antimicrobial peptides and also for the outer membrane integrity [60, 61].* eptA* and* arnT* genes belong to the BasS/R (PmrA/B) regulon. In *S*. Typhimurium, the activation of PhoP/Q upon depletion of Mg^2+^ and Ca^2+^ also leads to PmrA/B induction and thus allows integration of signals from different environmental cues. This cross talk between PmrA/B and PhoP/Q systems requires the adaptor protein PmrD [62]. The activation of the PhoP/Q system also induces transcription of* pagP* and* pagL* and hence upregulation of the encoded proteins, which acylate and deacylate lipid A, respectively [61] (Figure 4). However, PagP and PagL modification occurs posttranslationally after LPS is incorporated in the OM. Recently, it has been shown that even in* E*.* coli* PmrD can link the PhoP/Q and BasS/R two-component systems upon depletion of Mg^2+^ and promote lipid A modification by inducting the transcription of* eptA* and* arnT* genes [63].

Activation of BasS/R also induces the transcription of GcvB sRNA [64]. GcvB is relatively highly conserved, Hfq-dependent sRNA. It is the one of the most globally acting posttranscriptional regulators in bacteria, potentially regulating ~1% of all mRNAs in* Salmonella* and* E. coli* [65–67]. Its regulon is highly enriched with transporters of amino acids and short peptides, including the major ABC transporters Dpp and Opp. Its regulon members also include amino acid biosynthesis proteins and major transcription factors such as Lrp and PhoP. As PhoP is involved in lipid A modifications, translational repression of PhoP by GcvB [68] adds additional dimension to diverse inputs in modulating lipid A and LPS core modifications that could fine tune BasS/R and PhoP/Q induction. In* E*.* coli*, a deletion derivative of the* gcvB* strain was found to have increased expression of genes involved in the O-antigen biosynthesis like* rfbA*/*C*,* wbbH*,* wbbK,* and* wbbJ* [65]. Thus, MicA- and GcvB-dependent PhoP repression could jointly control lipid A and LPS modifications and, given the conservation of these sRNAs, they could play important roles in altering LPS and hence virulence phenotype.

## 6. Modification of Lipid A: Role of MgrR and MicA sRNAs

Several sRNAs play important modulatory roles for the modification systems of lipid A. For example, activation of PhoP/Q induces the transcription of the* mgrR* sRNA. MgrR has been shown to repress translation of the* eptB* mRNA [56]. The* eptB* gene is positively regulated at its transcriptional level by RpoE [57]. EptB transfers P-EtN to the second Kdo in the inner core and also contributes to resistance to antimicrobial peptides like polymyxin B [54, 56, 72]. In the integration of this signal transduction, PhoP/Q is repressed by MicA sRNA, thus linking RpoE and PhoP/Q in response to envelope stress. MicA is induced upon RpoE activation and represses* phoP* mRNA translation [21]. MicA base-pairs with the* phoP* mRNA in its translation initiation region and inhibits its translation by competing for ribosome binding [21] (Figures 2 and 4). PhoP translation is also repressed by base-pairing with GcvB sRNA [68]. However, this GcvB-mediated repression of* phoP* mRNA translation does not have an impact on MgrR sRNA [68].

Structural examination of LPS of a strain lacking WaaC heptosyltransferase I revealed that lipid A lacks P-EtN even under* eptA*-inducing conditions but preferentially incorporates P-EtN on the second Kdo in Ca^2+^-supplemented growth medium [54]. Under these conditions, RpoE is induced due to LPS defects, causing induction of the transcription of the* eptB* gene. Ca^2+^ can repress PhoP/Q, which turns off the synthesis of* mgrR* sRNA, whose transcription is PhoP/Q-dependent and at the same time allows the synthesis of EptB [56]. Ca^2+^ is also required for the enzymatic activity of EptB and thus enhances the incorporation of P-EtN on the second Kdo [72]. Lack of P-EtN in lipid A, as occurring in* waaC* mutants, can be explained by the preferred incorporation of P-EtN on the second Kdo rather than in lipid A, presumably to maintain a homeostatic control on such incorporation. However, in the absence of EptB, lipid A of* waaC* mutant exhibits nonstoichiometric incorporation of P-EtN [54].

**Figure 5:**
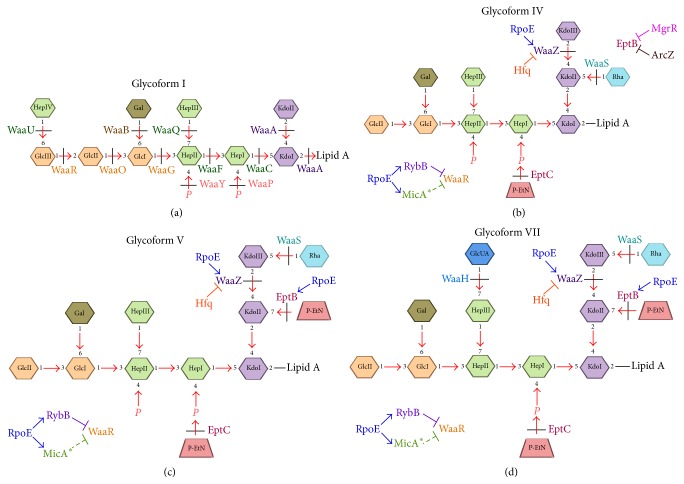
Schematic depiction of various LPS glycoforms observed in* E*.* coli* K-12. Glycoform I is the major LPS glycoform under nonstress condition, that is, in the absence of RpoE induction and other two-component systems (a). Induction of RpoE leads to the accumulation of glycoforms IV, V, and VII due to RybB- and MicA-mediated translational suppression of WaaR and induction of* waaZ* and* waaS* transcription that may also involve Hfq. In this process, RybB plays the central role. Glycoforms IV, V, and VII contain a third Kdo and rhamnose with a concomitant truncation in the outer core (b, c, and d). Glycoform IV accumulates when MgrR repression of* eptB* is predominant. The translation of the* eptB* mRNA is repressed by base-paring with MgrR under PhoP/Q-inducing conditions and also by ArcZ sRNA (b). When the RpoE induction is maximal, RpoE-driven transcription overrides MgrR-mediated repression of the EptB synthesis due to the hyperinduction of RpoE-regulated transcription of the* eptB* gene. Under such conditions, RybB at the same time represses translationally the WaaR synthesis (c). The glycoform VII is the major glycoform when RpoE-dependent* eptB* and* rybB* expression is induced with simultaneous induction of the* waaH* gene transcription leading to the GlcUA incorporation (d).

Activation of RpoE can override MgrR-mediated inhibition of* eptB* expression and hence promote incorporation of P-EtN on Kdo [54, 56, 57]. This can be attributed to MicA-dependent inhibition of PhoP under RpoE-inducing conditions as well as induction of strong RpoE-dependent transcriptional activation of the* eptB* gene [21]. As we will discuss further, P-EtN incorporation on the second Kdo can lead to further LPS structural alterations when RpoE is induced with intact LPS biosynthesis genes (Figure 5). An additional layer involving regulation of the* eptB* expression has revealed a role for ArcZ sRNA (Figure 4). Both ArcZ and MgrR are Hfq-dependent and inhibit translation and expression of* eptB* by base-pairing, although, in response to different environmental cues, ArcZ inhibits* eptB* expression in an ArcA/B-dependent manner, whereby phosphorylated ArcA represses ArcZ synthesis when oxygen concentrations are low [57]. Thus, EptB is controlled at several levels and explains its importance in contributing to LPS diversity and resistance to antibiotics like polymyxin B.

RpoE-regulated MicA sRNA is predicted to be involved in controlling phosphorylation of lipid A by regulating LpxT (Figure 4). MicA can base-pair with the* lpxT* mRNA and inhibit its translation [24]. Although its effect on the LPS structure has not been directly examined, structural analysis of LPS obtained from several different strains under simultaneous RpoE- and BasS/R-inducing conditions shows the absence of LpxT-dependent phosphorylation of lipid A, presumably due to the transcriptional induction of* micA*. Rather under such conditions, EptA-dependent P-EtN modification is observed and accumulation of triphosphorylated lipid A species does not occur [25]. However, the inhibition of LpxT activity upon BasS/R-inducing conditions is due to the expression of a short peptide PmrR, which has been shown to directly bind to LpxT [73] and hence constitutes posttranslational control (Figures 1 and 2). Thus, both BasS/R and MicA contribute to the inhibition of LpxT phosphorylation and allow incorporation of P-EtN and Ara4N in lipid A.

## 7. MicF and Regulation of Acylation

MicF sRNA is another regulatory* trans*-acting base-pairing RNA that controls lipid A modification by promoting the degradation of* lpxR* mRNA. LpxR is a lipid A deacylase with Ca^2+^-dependent 3′-O-deacylase activity and such modified lipid A is less bioactive [69, 74]. The* lpxR* gene is absent in* E. coli* K-12 but is found in the genomes of* E. coli* O157:H7,* Yersinia enterocolitica*,* Helicobacter pylori*, and* Vibrio cholera* [74]. MicF has been shown to bind to the* lpxR* mRNA within its coding region as well as in the ribosome-binding site [69]. The base-pairing of MicF within the coding sequence of the* lpxR* mRNA decreases its stability by rendering it susceptible to degradation by RNase E [69]. In* Y. enterocolitica* however,* lpxR* is negatively regulated by the PhoP/Q system and the regulator RovA [75]. As LpxR-mediated deacylation occurs after LPS translocation, the regulation of LpxR by MicF contributes to LPS modification event that occurs in the OM, expanding the role of sRNAs at various steps of the LPS biosynthesis and its modification.

## 8. Incorporation of the Third Kdo by WaaZ and Coordinated Repression of WaaR Synthesis: Regulation by RybB and MicA sRNAs

LPS is structurally highly heterogeneous, composed of several glycoforms that differ due to the incorporation of P-EtN on the second Kdo, incorporation of a third Kdo, incorporation of additional sugars like rhamnose, GlcN, uronic acids, and alterations in the numbers of phosphate residues in the LPS core. In most cases examined, the inner core has been found to contain an*α*-(2-4)-linked Kdo disaccharide, which is transferred by the bifunctional enzyme WaaA. Under normal laboratory growth conditions, the majority of LPS is composed of glycoform I and minor amounts of three additional glycoforms II, III, and IV can also be observed [29]. Glycoform I contains two Kdo residues in the inner core and four heptoses, and four hexoses attached in specific order in the inner core and the outer core (Figure 5). Thus far, 7 glycoforms have been structurally identified and extensively characterized [28]. Under RpoE-inducing conditions, LPS is primarily comprised of glycoform V and its derivatives [25]. This molecular switch is highly regulated and requires induction of the RpoE-transcribed genes* eptB*, sRNAs* micA,* and* rybB*, and the transcriptional upregulation of* waaZ* with a concomitant repression of WaaR synthesis. This switch is further amplified upon induction of the BasS/R and PhoB/R two-component systems [25].

Briefly,* waaZ* encodes a Kdo transferase required for the incorporation of the third Kdo [25]. The* waaS* gene encodes the rhamnosyl transferase and its incorporation requires prior addition of the third Kdo [25] (Figure 5). The WaaR glycosyltransferase mediates the incorporation of the third Glc in the outer core of LPS, which subsequently serves as an acceptor for the addition of the terminal heptose (HepIV) by WaaU (Figure 5). LPS species, defining glycoforms IV and V, have the same molecular masses but are structurally different. Glycoform IV is the most abundant LPS under PhoB/R and BasS/R induction and when RpoE-regulated* eptB* encoding P-EtN transferase is not induced. Under such conditions, EptB synthesis is silenced by PhoP/Q-dependent MgrR sRNA [56]. However, when RpoE is induced, for example, in the absence of the antisigma factor RseA or in the absence of Hfq, most LPS species are represented by glycoform V, which contains EptB-dependent P-EtN on the second Kdo [25]. P-EtN modification of the second Kdo favors the incorporation of rhamnose on the terminal third Kdo, which is otherwise attached to the second Kdo in glycoform IV. Thus, the preferential synthesis of glycoform V and its derivatives even when the EptB synthesis is repressed by MgrR sRNA suggests that RpoE induction overcomes silencing of the* eptB* expression and promotes this switch [25].

It is interesting that the glycoforms containing the third Kdo and rhamnose, such as glycoform IV and V, have a concomitant truncation of the terminal disaccharide as if WaaR is limiting [25] (Figure 5). Among several strains tested for their LPS composition, the absence of RseA antisigma factor that leads to maximal induction of RpoE causes near exclusive synthesis of glycoform V and VII or their derivatives. The molecular basis of this switch is explained by the induction of* eptB* expression and by two additional factors. Firstly, under such conditions, the* waaZ* transcription and WaaZ amounts are increased, promoting the incorporation of the third Kdo [25]. However, the transcription of the* waaZ* gene is not directly regulated from RpoE-recognized promoter and hence could occur after initiation of transcription in an Hfq-dependent manner. In agreement with this notion, a 3-fold increase in* waaZ* mRNA levels has been observed in an* hfq* mutant [76]. Secondly, the synthesis of WaaR is repressed preventing the addition of terminal disaccharide in the outer core, which is absent in such LPS structural forms. Thus, incorporation of the third Kdo and truncation in the outer core are highly coordinated processes and are orchestrated by RpoE-dependent sRNAs. This occurs due to the simultaneous induction of Hfq-dependent MicA and RybB sRNAs. Thus, introduction of a deletion of the* rybB* gene in strains lacking RseA restores the LPS synthesis to near normal levels and suppresses the synthesis of glycoforms that incorporate Kdo_3_Rha. A similar but partial restoration in LPS composition is also observed in the absence of MicA in strains lacking RseA. This is further supported by the findings that WaaR amounts are reduced in* rseA* mutants but restored to the normal levels when RybB is simultaneously removed. Consistent with repression of WaaR synthesis favoring the incorporation of LPS with the third Kdo,* waaR* mutants also synthesize LPS derivatives corresponding to glycoform IV without any requirement for RpoE induction.

*E*.* coli* preferentially synthesizes glycoform IV containing a third Kdo with truncation of the outer core disaccharide in PhoB/R-inducing conditions, without RpoE induction [25]. This switch from the usual glycoform I to IV can also be explained by increased transcription of the* rybB* sRNA [77]. This can provide an explanation based on the RybB-mediated repression of WaaR [25]. Thus, sRNAs control the LPS biosynthesis and its modifications at several levels. Regulation of the third Kdo incorporation is important, since this event prevents the incorporation of O-antigen as it is attached to the terminal heptose, which is absent in glycoforms IV and V. Further, Kdo residues form important contacts that are vital for TLR4-MD2 mediated immune response.

Pathogenic bacteria overcome the host defense in a variety of ways including subversion of function of dendritic cells (DC) that can interfere with the innate immune system. DCs express C-type lectin called DC-SIGN with which several pathogenic bacteria and even nonpathogenic* E*.* coli* interact through LPS. This interaction promotes adherence and phagocytosis and requires LPS core sugar residues [78]. Among several strains of* E. coli* with defects in the LPS core biogenesis, most significantly* waaR* mutants are resistant to phagocytosis by HeLa-DC-SIGN and the predicted ligand is either GlcIII or GlcNAc [78]. Since WaaR is the target of RybB sRNA and GlmY/GlmZ are required for synthesis of UDP-GlcNAc, this implies that LPS alterations by such sRNA-mediated interaction can play important role in bacterial adhesion and phagocytosis.

In summary, translational repression by PhoP/Q-regulated MgrR of* eptB *expression and repression of WaaR synthesis by RybB promotes the synthesis of glycoform IV. However, induction of RpoE leads to increased transcription of* waaZ*,* eptB*, and* rybB*. This leads to switch causing synthesis of glycoform V. Further, BasS/R activation under RpoE-inducing conditions amplifies this incorporation of glycoforms with a third Kdo and rhamnose due to increased expression of* waaS*. Thus, RpoE, PhoP/Q, BasS/R, and PhoB/R jointly control LPS composition using a network of sRNA-mediated control (Figures 1, 2, and 5).

## 9. Impact of Regulated LPS Modifications on Virulence Associated Phenotypes

Deletion of PhoP/Q-regulated MgrR results in a 10-fold increase in the resistance to polymyxin B. PhoP/Q and PmrA/B are both activated following endocytosis of live* S*. Typhimurium cells by RAW 264.7 macrophage tumour cells, resulting in multiple partial covalent modifications of lipid A [79]. Addition of the l-Ara4N and palmitate residues to lipid A confers resistance to polymyxins and *β*-defensins, respectively [80, 81]. Additionally, competitive infection experiments in mouse models of* S*. Typhimurium infection showed a decrease in survival of mutants unable to incorporate P-EtN when compared to wild-type strains [82]. RybB-mediated repression of WaaR could as well contribute to bacterial adhesion and phagocytosis as discussed earlier. Severe defects in LPS or lack of PhoP have been shown to cause defects in colonization, biofilm formation, and sensitivity to antimicrobial peptides in many pathogenic bacteria such as* Yersinia pestis* [83].

## 10. Other Targets of sRNAs in LPS Biosynthesis

In* Salmonella*, it has been reported that in the absence of Hfq, several genes, whose products are involved in lipid A (*lpxA* and* lpxD*), phospholipids (*fabZ*), and O-antigen biogenesis (*rfbACKMJU*) are upregulated [84]. It will be interesting to identify if any specific sRNA is involved in this process. Some LPS alterations have also been reported in an* E*.* coli* UPEC variant lacking* hfq* [27]. Expression of* lpxC*,* ugd,* and* waaH* has been shown to be upregulated along with the concomitant upregulation of GcvB, RybB, and GadY sRNAs following phosphate starvation [28, 77].

Alterations in LPS profiles have been reported in strains lacking CsrA or upon its overexpression [85]. CsrA is a RNA-binding protein and was initially identified as a regulator of glycogen biosynthesis [86]. CsrA binds at GGA-rich motifs and could promote RNA decay [87]. The activity of CsrA is regulated by specific sRNAs (CsrB/CsrC).* Shigella* strains lacking CsrA also have been shown to have altered LPS profiles [88]. Similarly, transcriptome analysis of strain lacking YbeY RNase revealed that* eptA*,* lpxM,* and* rfaH* could be regulated by RdlA, RyeA, and RyjB sRNAs, respectively [71]. YbeY has been shown to regulate the expression of some sRNAs [71]. Further LPS structural analysis will be required to draw a direct conclusion of the involvement of these additional sRNAs in the control of LPS biosynthesis and its modifications. In the pathogenic bacterium* Porphyromonas gingivalis*, the synthesis of LPS was found to be altered by deletion of antisense RNA molecule located within a 77-bp inverted repeat element. This element lies in the 5′ region of the K-antigen synthesis locus [89].

Considering the involvement of other sRNAs, it is tempting to speculate that depletion of sugar nucleotides pools could contribute to structural heterogeneity. Thus, Spot42-dependent repression of GalK could limit precursor UDP-Glc and UDP-Gal availability, when CRP is activated [90]. There is precedence for such a signal transduction conferring phenotype that can be attributed to reduction in the availability of UDP-Glc pools. A derivative of enterohemorrhagic* E*.* coli* O157:H7 with* lpxM* mutation was found to have a truncation in LPS due to limiting amounts of UDP-Glc precursor. This defect could be complemented by the overexpression of* galU* [91]. A similar scenario can be imagined if the synthesis of sugar transports is impaired, which require SgrS sRNA. sRNA-dependent LPS heterogeneity and truncation of LPS has also been shown when GadY sRNA is overexpressed in a* waaY* mutant, while studying role of LPS in biofilm formation [70]. This truncation has been ascribed to reduction in the expression of* waaQ* operon. Further work will be required to know exact molecular basis of GadY control of expression of the* waaQ* operon.

## 11. Conclusion

It is now abundantly clear that sRNAs play important roles in gene expression and regulate several functions. Until recently, the main function of MicA and RybB sRNA was thought to be translational repression of OMP biosynthesis. Both of these sRNAs are regulated by the RpoE sigma factor. RpoE controls the expression of genes involved in the cell envelope biogenesis in response to changes in OMP and LPS composition. Here, we have highlighted that MicA and RybB also control the LPS biogenesis and its modifications. Further, the newly identified RpoE-regulated sRNA SlrA controls the expression of the most abundant protein Lpp and negatively regulates RpoE expression. Together, these three sRNAs monitor LPS and the OM composition. As LPS is highly heterogeneous and additional sRNAs have been predicted to regulate the LPS biosynthesis or its modifications, it will be interesting to examine LPS composition of strains lacking these sRNAs or when they are overexpressed. Also, it will be pertinent to examine how RybB mediates the translational repression of WaaR, an enzyme that is key to the synthesis of different glycoforms. The chemical structure of such novel glycoforms should be resolved and their inability to elicit or escape immune response should be considered carefully. In addition to the PhoP/Q and BasS/R two-component systems, PhoB/R also regulates the incorporation of glucuronic acid and P-EtN in the inner core. Microarray studies have revealed that expression of several sRNAs is also altered by the PhoB/R-regulated response upon phosphate starvation. Further research is required to address if these PhoB/R-regulated sRNAs impact the structure and composition of LPS. In summary, we can conclude that several sRNAs regulate LPS composition and contribute to its structural diversity at several steps.

## Figures and Tables

**Table 1:** Known and predicted sRNAs involved in LPS biosynthesis and its modifications.

sRNA	Target	Effect on LPS	Reference(s)
GlmY/GlmZ	GlmS	GlcN6P precursor of LPS	[35, 39, 40]
MicA	Repression of PhoP/Q	P-EtN addition to the second Kdo	[21, 25]
	Repression of WaaR	Switch between glycoforms IV and V	[25]
	Repression of LpxT	Incorporation of lipid A modifications	[24]
GcvB	Repression of PhoP/Q	Direct effect on LPS not known	[68]
RybB	Repression of WaaR	Major control of the synthesis of glycoforms with truncation in the outer core and incorporation of the third Kdo	[25]
SlrA	Repression of Lpp	Levels of fatty acid (phospholipids vs LPS) control	[9, 22]
MgrR	Repression of EptB	Repression of P-EtN incorporation on the second Kdo	[25, 56]
ArcZ	Repression of EptB	Impact on LPS not known	[57]
MicF	LpxR	Deacylation of lipid A	[69]
GadY	*waaQ* operon	Overexpression causes transcriptional reduction, direct effect on transcription duo to base-pairing not know	[70]
RyeA	LpxM	Predicted from microarray data	[71]
RdlA	EptA	Predicted from microarray data	[71]
RyjB	RfaH	Predicted from microarray data	[71]
